# An Automatic Detection and Localization of Mammographic Microcalcifications ROI with Multi-Scale Features Using the Radiomics Analysis Approach

**DOI:** 10.3390/cancers13235916

**Published:** 2021-11-24

**Authors:** Tariq Mahmood, Jianqiang Li, Yan Pei, Faheem Akhtar, Azhar Imran, Muhammad Yaqub

**Affiliations:** 1 The School of Software Engineering, Beijing University of Technology, Beijing 100024, China; tmsherazi@ue.edu.pk (T.M.); lijianqiang@bjut.edu.cn (J.L.); myaqubciitswl@gmail.com (M.Y.); 2 Division of Science and Technology, University of Education, Lahore 54000, Pakistan; 3 Beijing Engineering Research Center for IoT Software and Systems, Beijing 100124, China; 4 Computer Science Division, University of Aizu, Aizuwakamatsu 965-8580, Japan; 5 Department of Computer Science, Sukkur IBA University, Sukkur 65200, Pakistan; fahim.akhtar@iba-suk.edu.pk; 6 Department of Creative Technologies, Air University, Islamabad 44000, Pakistan; azhar.imran@mail.au.edu.pk

**Keywords:** microcalcification, radiomics approach, support vector machine, data-augmentation, computer aided diagnosis

## Abstract

**Simple Summary:**

Breast cancer is one of the foremost causes of cancer-related mortality in women. It is curable and controllable only if detected early. Microcalcifications in breast tissue are essential predictors for radiologists to detect early-stage breast cancer. This study proposes a method for detecting and classifying microcalcifications in mammogram images to predict breast lesions, using machine learning coupled with an interpretable radiomics approach. The method was evaluated using a publicly accessible dataset, which may aid radiologists and clinicians in identifying breast cancer in their regular clinical practices. This study contributes to the field of predictive modeling in healthcare.

**Abstract:**

Microcalcifications in breast tissue can be an early sign of breast cancer, and play a crucial role in breast cancer screening. This study proposes a radiomics approach based on advanced machine learning algorithms for diagnosing pathological microcalcifications in mammogram images and provides radiologists with a valuable decision support system (in regard to diagnosing patients). An adaptive enhancement method based on the contourlet transform is proposed to enhance microcalcifications and effectively suppress background and noise. Textural and statistical features are extracted from each wavelet layer’s high-frequency coefficients to detect microcalcification regions. The top-hat morphological operator and wavelet transform segment microcalcifications, implying their exact locations. Finally, the proposed radiomic fusion algorithm is employed to classify the selected features into benign and malignant. The proposed model’s diagnostic performance was evaluated on the MIAS dataset and compared with traditional machine learning models, such as the support vector machine, K-nearest neighbor, and random forest, using different evaluation parameters. Our proposed approach outperformed existing models in diagnosing microcalcification by achieving an 0.90 area under the curve, 0.98 sensitivity, and 0.98 accuracy. The experimental findings concur with expert observations, indicating that the proposed approach is most effective and practical for early diagnosing breast microcalcifications, substantially improving the work efficiency of physicians.

## 1. Introduction

Breast cancer is the most common malignancy in women across the globe [1]. Different medical imaging modalities, primarily mammograms, are the preferred diagnostic tools for early breast cancer screening, and have been proven to decrease the mortality rate by up to 30% [2]. Mammography detects about up to 80–90% of breast cancer patients without symptoms at an initial stage [3]. The Breast Imaging Reporting and Data System (BI-RADS) lexicon characterizes mammogram features by breast density, mass, calcification, asymmetry, lesion location, and related findings [4]. A mammogram distinguishes the breast density based on the recommended lexicon: fatty, scattered, heterogeneously dense, and highly dense. On a mammogram, benign calcifications are often classified as a large rod, vascular, coarse, and popcorn. In comparison, suspicious calcifications in the breast are characterized as diffuse regional, linear, clustered, amorphous, and segmental [5]. The BI-RADS lexicon distinguishes calcifications as benign or malignant based on their shape, density, and distribution inside the breast, particularly the location to the nipple region.

Breast microcalcification (MC) is an indicator of potential carcinoma; it has been the only detectable sign of nonpalpable breast cancer during screening (25–43% clinically; 62–98% ductal carcinoma in situ (DCIS)) [6]. MCs are small glandular bright calcium deposits that typically range between 0.01 and 0.1 mm in size, and appear as a collection of regionalized high-intensity areas on radiology film [7]. Clinical studies indicate that there are 5 or more MC points per centimeter [8]. MC is, most likely, intramammary, and located within and around ducts, lobules, and vascular structures [9]. Size, morphology, number, the pattern of distribution, locality, and density, among other characteristics, contribute to determining pathology [10]. Radiologists interpret mammograms to recognize benign breast calcifications that do not need a biopsy, and to avoid unnecessary treatments, and reduce patient anxiety. Mammogram anomalies with radiological scores of 4 on the BI-RADS are considered “highly dubious” for malignancy. Thus, a pathological investigation is required to confirm the results as either benign or malignant for further surgical or non-invasive plans. On the other hand, real-world clinical statistics indicate that about 35% of malignant MCs required mastectomies, among all of the biopsied specimens that subsequently developed breast cancer [11,12].

According to the BI-RADS lexicon, a biopsy is strongly advised for suspected MC, categorized as BI-RADS category 4, to ascertain its pathological nature [13]. Moreover, the doctor examines a large volume of medical images regularly, resulting in visual fatigue [14]. Double reading mammograms have improved diagnostic sensitivity and detection of breast lesions by 3 to 15%, but exacerbated the current shortage of skilled radiologists performing large-scale routine screenings [15,16]. Thus, a more precise and unbiased approach for the BI-RADS 4 mammographic MC assessment is required to predict cancerous growths for prompt treatment, or benign prediction, for a potential downgrading classification and observed up-approach.

Several studies have reported on various approaches to detect and classify MC in digital mammograms, i.e., as benign or malignant [17,18]. Although many methods for identifying MC points have been proposed, each has a high incidence of false positives [19]. Radiomics is a novel image analysis technique that enables the quantitative detection of complex features in radiographic images that represent the heterogeneity of the texture, shape, and size of various tissue phenotypes associated with multiple clinical outcomes [12]. The radiomics approach was proven to render automated computer-aided classifiers of radiological images coupled with machine learning algorithms. Radiomics analyses have attracted much attention in clinical applications in the last few years, showing convincing results in clinical decision support methods and advancing fully customized therapeutic decision-making (i.e., regarding prognosis and prediction) [20]. Radiomics analysis of a mammogram is assumed to capture and quantify the morphological and textural heterogeneity features of MC, invisible to the radiologist’s eyes [21]. Texture analysis has proven helpful in detecting and categorizing lesions by employing a set of local statistical features of pixel intensity. Radiomics potentially overcomes the identified problems, and enhances breast cancer diagnosis performance in patients recalled for screening after calcifications are detected during a diagnostic mammogram.

Thus, this study proposes a radiomics approach based on advanced machine learning algorithms for quick and precise diagnosing of MC regions in mammogram images. First, each image was denoised and enhanced using various techniques to eliminate background noise, artifacts, and to improve image quality. Afterward, the top-hat morphologic operator and wavelet transform were employed to recognize the seed points and determine the suspicious region. Suspected textural and statistical features of MCs were retrieved using the wavelet analysis and the mathematical morphology approach in the mammogram images. Finally, we combined the aggregate features and fed them into a proposed model and a support vector machine (SVM) to train and classify the extracted regions of interest (ROIs) of the mammogram images. The radiomic model’s diagnostic performance was tested and compared with conventional algorithms, such as the SVM [22], K-nearest neighbor (K-NN) [23], and random forest (RF) [24], using various evaluation parameters. The finding is congruent with the perceptions of doctors, indicating that the proposed approach detects MC more precisely and realistically than earlier techniques. It enables physicians to work more efficiently by collaborating with a computer-aided diagnostics system that assists clinicians in early recognition and improves effectiveness.

The paper is structured as follows: Section 2 reviews the available literature associated with diagnostic mammography. The proposed methodology, image preprocessing, and MC ROI detection and classification approaches are presented in Section 3. The experimental findings of the proposed work, using various performance parameters, are compared and shown in Section 4. Section 5 discusses the experimental results. Finally, Section 6 concludes the study’s findings.

## 2. Related Works

Machine learning, particularly deep learning (DL), is receiving more interest due to its ability to perform automated image analysis, and improve breast cancer screening, prediction accuracy, and efficiency. Consequently, researchers have made significant contributions to the development and improvement of computer-aided design systems. Doctors employ computer-aided diagnostic (CAD) systems to identify breast cancer; these systems boost accuracy by more than 10% despite the system’s limited capacity to detect dense breast tumors [25].

### 2.1. Mammography Characterization through Conventional Machine Learning Models

Several machine learning techniques were employed to categorize MC spots to diagnose breast cancer, including SVM, K-NN, Naive Bayes, and RF. Manual feature extraction approaches are computationally inefficient and decrease the model performance of machine learning-based CAD systems. Martins et al. [26] designed a technique for diagnosing breast lesions using mammogram images. Segmentation of breast lesions was conducted using the K-means algorithm; textural features were then extracted from the segmented images. The obtained features were categorized employing the SVM algorithm, which achieved an accuracy of 0.85 on the digital database for screening mammogram (DDSM). Loizidou et al. [27] obtained 0.99 accuracy in automatically identifying and categorizing the MC cluster using a temporally sequential subtraction strategy based on SVM. Gaikwad et al. [28] used SVM to detect MC by combining linear kernel functions and polynomial kernels. Chakraborty et al. [29] provided a model for detecting and classifying breast masses by incorporating Bayesian, artificial neural network, and low- to high-level intensity thresholding. Liu et al. [30] presented a unique technique coupled with a simulated annealing genetic algorithm for feature selection; a constraint-sensitive SVM for classification resulted in a 0.95 accuracy and a 2.2 misdiagnosis rate. Elmoufidi et al. [31] built a fully automatic CAD system to segment and classify the mammogram’s ROI as malignant or benign using multiple-instance learning (MIL) algorithms and achieved 0.95 sensitivity, at a false-positive per image (FPi) 2.6 on DDSM, and 0.94 sensitivity at an FPi 2.9 on MIAS, which is beneficial for decision sustenance of radiologists. Beham et al. [23] used wavelet transform to extract local binary patterns. The K-NN algorithm classified the collected features into benign and malignant classes with an accuracy of 0.73. Obaidullah et al. [24] developed an image descriptor that outperformed the histogram of oriented gradient and a convolutional neural network for breast lesion classification with a 0.98 AUC value without employing clinical information.

### 2.2. Mammography Characterization through Conventional Deep Learning Models

The research community has made various attempts to improve the clinical performances of radiologists by developing CAD systems based on deep learning to identify breast masses detected during mammogram screening. Numerous investigations have led to the development of various mammogram-based detection systems, which are briefly discussed. Cai et al. [18] developed a deep CNN model for automated characterization of microcalcification and classified them based on the extracted automatic and handmade features. The proposed model attained an accuracy of 0.89 and a sensitivity of 0.86 using 749 private mammogram images. Wang et al. [32] designed a fused network comprised of deep neural networks, and extreme learning machines (ELMs) was employed to identify and classify breast cancers based on fused properties, such as morphology, texture, and density, with a classification accuracy of 0.80. Soulami et al. [33] demonstrated an excellent approach for removing pectoral muscle using entropy thresholding and an ROI segmentation using particle swarm optimization (PSO). SVM algorithms perform better with an accuracy of 0.83 in categorizing the extracted ROI into normal and abnormal properties. Guan et al. [34] employed an ROI-based technique in combination with an N-Net deep convolutional neural network to identify asymmetry patterns related to breast cancer diagnosis in digital mammograms.

Rouhi et al. [35] proposed two hybrid algorithms for breast tumor identification that incorporate region-based, contour-based, and clustering segmentation techniques. Different classifiers, such as SVM, K-NN, multilayer perceptron (MLP), and linear discriminant analysis (LDA), were used to classify tumors as benign or malignant with sensitivity and accuracy, of 0.83 and 0.89, respectively. Rizzi et al. [36]—two-level wavelet decomposition was used to automate identification and localization of MC clusters. Singularity points were extracted from the reconstructed image using another wavelet, and each level of decomposition was handled using hard thresholds. Yu et al. [37] developed a CAD system, which contained two phases for automatic detection of MC clusters using neural network classifiers. In the first phase, mixed features segregated probable MC pixels, and in the second phase, 31 statistical characteristics validated potential objects. The findings indicate that the combination of model-based and statistical textural features effectively classify MC, with a detection rate of 0.94, and an FPi of 1.0 per image. Papadopoulosa et al. [38] implemented morphological filtering and neural networks for automatic ROI extraction. The proposed method can effectively get ROI; however, morphological filtering and thresholding may ignore smaller regions of interest. Elter et al. [39] applied an independent component analysis to ROI feature extraction and used an artificial neural network to classify the model to identify the ROI.

There is a need for an approach that uses textural indicators to detect and differentiate clustered MC from normal breast tissue while minimizing bias. Our study proposes a strategy for identifying the MC region based on wavelet feature architecture. We evaluated and verified using feature vectors generated by morphological top-hat filters. Moreover, this research used a radiomics analysis strategy to classify MC areas, surpassing existing approaches with a 0.98 accuracy, 0.90 AUC, and 0.98 sensitivity, with 1.2 FPi.

## 3. Materials and Methods

**Figure 1:**
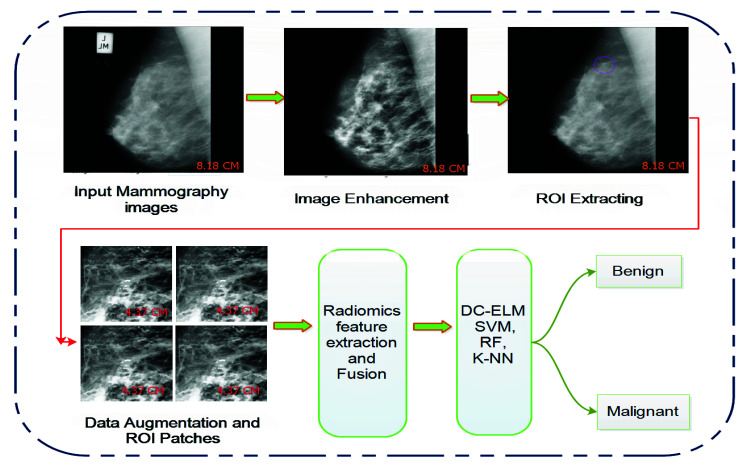
Step-wise illustration of the proposed classification framework consists of mammogram preprocessing, MC ROI detection, lesion segmentations, training of machine learning classifier to distinguish MC ROI into benign and malignant classes.

Early diagnosis of microcalcification increases a patient’s survival chances and helps doctors discriminate benign and malignant tissues during the curing process. The raw mammogram images influenced by inherent noise and illumination imperfections require extensive clarity, denoising, and normalization. This study focuses on MC diagnosing, which uses radiomics analysis concepts to evaluate the input features of a particular ROI. Our suggested study pertains to four steps: mammogram preprocessing and data augmentation, suspicious region detection and extraction, ROI feature learning, and training and testing of deep learning and machine learning approaches to classify the MC cluster. Initially, all images were preprocessed to remove noise and enhance image quality using various image denoising filters and enhancement algorithms. Afterward, ROI was defined for feature extraction using mathematical morphology and wavelet analysis. Wavelet transformations effectively evaluate the original image by reducing the gap between the least mean square error and the noise in the predicted and input images. The proposed architecture of our model for mammogram classification based on MC detection regions is explanted in Figure 1.

The significant contribution of this study is:

- We created an efficient strategy for identifying and categorizing MC mammograms using a radiomics analysis approach. It is easy to implement, convincing, well-defined, and overcomes small and large feature search space optimization issues.
- The optimum feature collections from the input data were achieved using wavelet analysis, which suppresses redundant and superfluous characteristics and prioritizes feature significance.
- This study combined wavelet transform and the top-hat operator to filter out seed regions of calcification spots, resulting in a significant reduction in false detection and improved detection accuracy.
- The effectiveness of the proposed feature learning model is compared to that of existing strategies. The mammogram dataset was validated using well-established clinical validation techniques.

### 3.1. Dataset

Due to confidentiality concerns, obtaining real-time mammogram images for research purposes is challenging. Each image used in this study was obtained from the Mammographic Image Analysis Society (MIAS [40]) database to evaluate the proposed model’s effectiveness. Due to many missing data points, noise, and variable image sizes, the resulting dataset contained low-quality images with a high proportion of false-positives and -negatives. The MIAS dataset contained 322 digital mammograms of 161 patients, including many breast views and correct annotations evaluated for breast anomalies by a radiologist. Four mammograms were collected for every patient in the mediolateral oblique (MLO) view and craniocaudal (CC) view.

**Figure 2:**
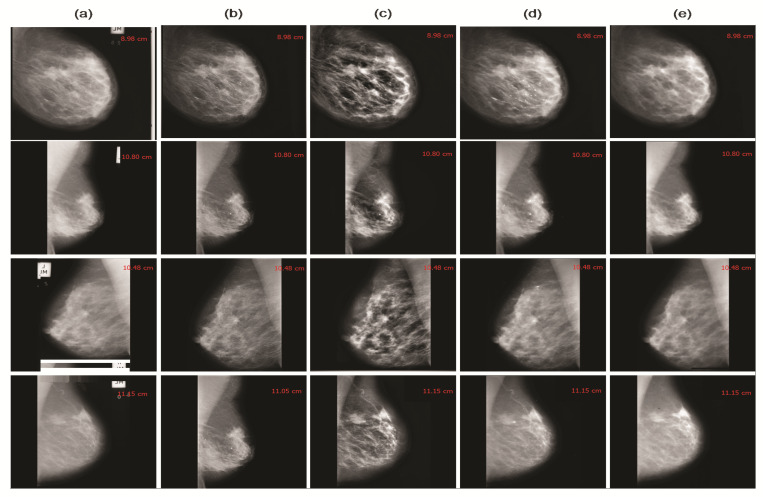
Represents the image preprocessing process to enhancement the image contrast and remove the noise, label, and unnecessary edges, (**a**) original raw mammogram image with calcification, (**b**) adaptive unsharp masking, (**c**) image enhancement (CLAHE), (**d**) dilation, (**e**) erosion.

Each breast has two projections (left and right), one for each instance, as illustrated in Figure 2a. The obtained dataset includes 54 malignant cases, 67 benign cases, and 201 normal cases, validated and diagnosed using the ground truth data of doctors. All images are grayscale and have 1024×1024 pixels, and a 200×200 mm resolution in PGM format, including calcification anomalies and pathologic ground truth concerning diagnosis. Each image was reduced to a predetermined scale of 224×224 using nearest-neighbor integration to train and validate the proposed model for MC classification. The proposed study used 968 images split into training and validation sets, as described in Table 1. The training set contained 775 images (80% of the dataset), and the validation set comprised 193 images (20 % of the dataset) to assess the trained model’s accuracy. The retrieved properties are used to construct algorithms for benign and malignant tumor classification.

### 3.2. Image Preprocessing and Data Augmentation

Image preprocessing improves image quality, making it more acceptable for visual features used by human and computer recognition systems. The type of noise observed is background noise, tape artifacts, high-intensity rectangular label, edge shadowing effect, and low-intensity label in mammogram images, which may impair the identification’s efficacy. The mammographic image has low contrast due to the intricacy and heterogeneity of breast tissue; yet, the doctor analyzes the breast image for limited information [41]. Even experienced physicians have difficulty detecting concealed MC, resulting in misdiagnoses [3,42]. In this study, we employed distinct preprocessing paradigms to improve the appearance of breast images by smoothing, brightening, denoising, and detecting edges [43]. We used noise reduction methods, such as adaptive median, Gaussian, and bilateral filtering to efficiently reduce noise while retaining sharp edges. They efficiently suppress impulse noise, speckle noise, and salt and pepper noise. After denoising, the artifact and pectoral muscle were removed, which occupied 70% of the mammogram’s surface, with the remaining 30% of the cover devoted solely to the breast. In the non-breast area, tissue density was strongly linked, which may influence subsequent mammogram analysis [44].

**Figure 3:**
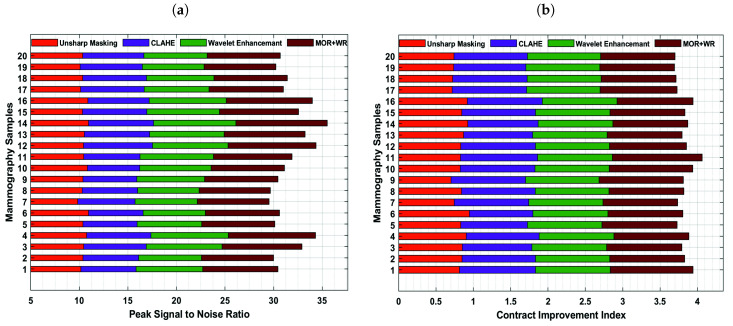
The figures represent: (**a**) the peak signal-to-noise ratio reflects the denoising effect of the mammogram. The higher the value, the more effective the denoising impact. (**b**) The image contrast enhancement index quantifies the degree to which the edges of a mammogram have been improved. The higher the value, the more effective the augmentation of the mammogram.

This study employed image enhancement approaches based on adaptive unsharp masking, contrast limited adaptive histogram equalization (CLAHE), and wavelet analysis, to improve the image’s quality and smoothness, as shown in Figure 2a–e. The proposed techniques emphasize MC points while reducing the redundant background information and enhancing the image’s weak edges and calcification areas. The suitable image enhancement methods are selected according to the actual needs to boost the image’s contrast and achieve the mammogram image enhancement effect. This study used the contrast improvement index (CII) for accurate evaluation, reflecting the effect of image edge enhancement and the peak signal-to-noise ratio (PSNR) value, indicating the image’s denoising to verify the method’s effectiveness. Moreover, we measured the CII and PSNR of 20 mammogram images to evaluate their enhanced performance. The higher the PSNR and CII values, signifying a more significant denoising and enhancement impact. The adaptive unsharp masking and CLAHE techniques are ineffective at improving MC areas. Figure 3a,b shows that the combined method, based on the morphological top-hat operator (MOR) and wavelet reconstruction (WR), performs better than unsharp masking and CLAHE.

Furthermore, in this study, we artificially inflated the dataset using distinctive data augmentation paradigms [45], such as scaling, flipping, contrast, and rotation to improve robustness and detection accuracy, as shown in Table 2. The mammogram images are rotated in different degrees to increase the dataset’s size and mitigate overfitting across each training epoch. As a result, this study augmented 968 mammogram images, which included 536 benign and 346 malignant instances.

### 3.3. Wavelet Analysis

**Figure 4:**
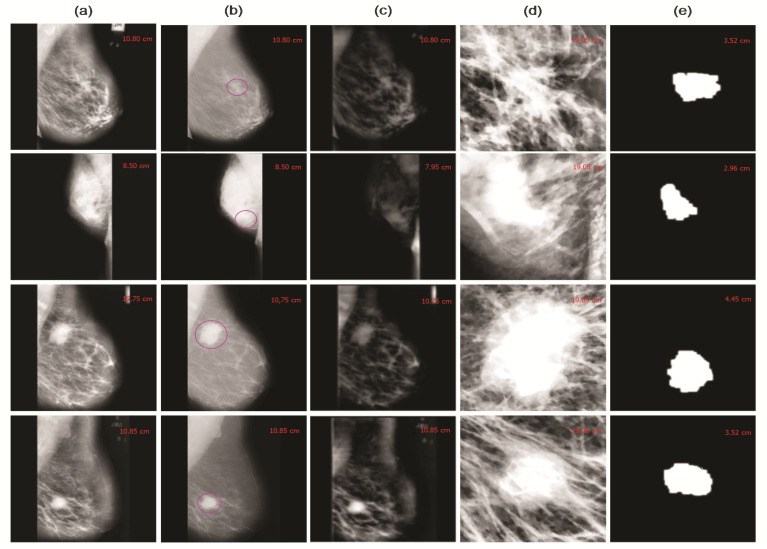
An illustrative example, conferring segmentation of suspected MCs on mammogram images of the right and left breasts with fibrocystic anomalies. (**a**) Original image with calcification; (**b**) concentrated MC (denoted by a circle) seems weak compared to the thick background in the MLO and CC view; (**c**) top-hat transform; (**d**) enlarged view of the suspected MC region; (**e**) segmented MC mask detected is used to classify the characteristics.

The image’s calcification points are discrete points interspersed in the low-frequency background, and extremely high-frequency noise in the image [46]. In this study, we used the wavelet analysis to localize and extract the MC ROI from the calcium regions in the mammogram, as shown in Figure 4a–e. Moreover, the wavelet transform eliminates low-frequency noise from the image, revealing only high-frequency information, such as calcification patches. The wavelet transform and the high-frequency coefficients of each layer’s wavelet are used to extract the radiomic texture feature, using the gray level co-occurrence matrix approach (GLCM), morphological feature, and intensity histogram features. The different layers of wavelet coefficients are illustrated in Equation (1).

(1) $$g=D+V_{k}^{i}$$

In Equation (1), $V_{k}^{i}$ depicts the high-frequency coefficient of each layer obtained by decomposition; *D* depicts the low-frequency coefficient obtained by decomposition; k=1,2,3,4 are the different decomposition layers; *i* are the different decomposition directions, which are horizontal, vertical, and diagonal. The results indicate that the microcalcification point coefficients are dispersed mainly on the second and third layer high-frequency wavelet decomposition coefficients selected for reconstruction, resulting in a noise-free breast image. The aspects used to evaluate a wavelet function’s quality include orthogonality, support, symmetry, vanishing moment, and regularity.

### 3.4. Mathematical Morphology

Mathematical morphology has achieved great success in visual inspection, robot vision, and medical image processing applications. The core idea of mathematical morphology is to segment the weak and tiny calcification points using specific morphological structural components [47,48]. However, this study employed mathematical morphology to detect calcified spots and segments from the mammogram background. Furthermore, this study used the region growing approach, which interprets neighboring pixels as new seed points by combining them with a sequence of seed pixels. The appropriate seed points were selected automatically based on regional shape, regional gray distribution rules, and regional gray differences.

### 3.5. Top-Hat Algorithm

This study proposed a top-hat algorithm to integrate the fundamental operations of grayscale mathematical morphology [49]. It conducts coarse segmentation of the image’s MC points and extracts of micro-calcification. The top-hat operator has high-pass filtering features, enabling the target to be extracted from a complex background by selecting appropriate structural elements, as depicted in Figure 4c. The primary function of the top-hat operator is to increase visual contrast by emphasizing edge information and reduce noise [50]. Equation (2) depicts the definition of the top-hot operator.

(2) $$t_{h}\left(K\right)=g-g\circ f$$

where *g*∘*f* is an open operation in morphology that eliminates small image elements in the mammogram and smooths the boundaries to achieve edge enhancement. By subtracting the original image *g* from the image processed by *g*∘*f*, most of the image’s background feature is removed, retaining the tiny image area with a high application value. The definition of the closed top-hat operator is shown as Equation (3).

(3) $$t_{h}\left(B\right)=g-g•f$$

where *g*•*f* is a closed operation in morphology. It smooths the image’s borders and eliminates the majority of the image.

### 3.6. Radiomics Based Proposed Method

This study develops a method for detecting and classifying MC in mammogram images to predict breast lesions using machine learning and an interpretable radiomics approach. It combines the abstraction capabilities of deep convolutional neural networks with the performance of an extreme learning machine (DC-ELM) [51,52]. The proposed architecture comprises an input layer, an output layer, and several hidden layers that configure alternately, as one convolution layer supported by one pooling layer [18,33]. The convolution layer is constructed from a collection of feature maps that are connected through convolution nodes. The data weights of identical feature maps are shared while remaining unique across maps.

The proposed model yields higher features, layer-by-layer, throughout the convolution feature abstracting training phase. Multiple hidden convolutions and pooling layers effectively extract the high-level characteristics required for classification tasks. The node‘s feature map with the convolution layer is combined with all other feature maps. In contrast, the node‘s feature map with the pooling layer is connected to a single corresponding feature map in the preceding convolution layer. The final pooling layer employs stochastic pooling, which significantly mitigates the feature map‘s size, providing the full connection with the output layer. Afterward, the model is trained using an arbitrary weight distribution for each hidden node. The weight values are changed by using the optimization method. The model’s generalization characteristic relies on the input weights and bias, and the random initialization of these parameters presents significant challenges. The generality of the model is enhanced because the hidden layer biases and input weights may be adjusted freely if the activation function is separable arbitrarily. Finally, the output weight and ground truth labels of MC ROI images may be computed to perform the training.

### 3.7. Standard Classifiers

SVM is a machine learning method based on the structural risk minimization theory, which has an advantage in pattern recognition, regression analysis, and other fields [53]. SVM classifiers aim to construct a hyperplane to offer an efficient and accurate method for diagnosing and categorizing mammogram images. It is often employed when training datasets that are inadequate, to obtain more generalization and to overcome linear and non-linear constraints on separating data points of each class [22,33,35]. It makes an effort to minimize the percentage of nonzero weights and problems with overfitting. The basic idea of an SVM is to map inseparable vectors to one high-dimensional feature space and find the optimal classification hyperplane in the high-dimensional feature space. To verify our suggested strategy, we executed a classification task, using a support vector machine algorithm. A radiomics analysis approach was performed to detect microcalcification ROIs and extract features to provide input for SVM and other machine learning algorithms. We classify MC ROIs using the support vector machine algorithm’s non-linear kernel function. It is observed that the support vector machine classifier provides more inclusive results on a limited number of datasets.

In this research, we used K-NN supervised machine learning algorithms for binary classification to examine and assess the achievement of our framework [35]. The microcalcification ROI detection and image segmentation methods used in this work are the same as employed in the SVM algorithm. We set the maximum k value with an optimal error rate to avoid the K-NN classifier from overfitting. Ali et al. [54] used wavelet transformations to extract features from a mammogram image and classified the extracted features into benign and malignant instances using the K-NN algorithm. The findings indicate that the K-NN classifier’s performance was insufficient for classifying breast cancer microcalcification ROI using a mammogram image.

Random forest is a kind of supervised machine learning model employed in this study to categorize microcalcification regions. The RF algorithm uses a majority vote to perform classification problems. Random forest performance was compared to other suggested approaches, and it was determined that random forest performed well. Fadil et al. [55] developed a computer-based automated approach for detecting and segmenting the MC regions in mammogram images using the discrete wavelet transform and random forest. The results suggest that RF achieved an accuracy of 0.83 and a sensitivity of 0.78.

### 3.8. Performance Measures

Our proposed scheme improved model accuracy significantly by classifying detected MC ROIs into malignant and benign instances. The suggested method’s performance was measured using the public MIAS database. Evaluation metrics, such as accuracy, F-score, recall, precision, sensitivity, specificity, and area under the curve (AUC), were used to analyze the presented model’s performance. The following formulas are used to calculate the performance parameter.

**Accuracy** is defined as the ratio of correctly identified images to total images.

(4) $$\begin{array}{c} ACC\left(\%\right)=\frac{Tp+Tn}{Tp+Fp+Fn+Tn} \end{array}$$

In Equation (4), *Tp* is true positive, *Tn* is true negative, *Fp* is false positive, and *Fn* is false negative rates.

**F-score** is the harmonic mean of precision and recall as shown in Equation (5), which is used independently to determine the correctness of test datasets.

(5) $$\begin{array}{c} F-score\left(\%\right)=2\times \frac{\frac{TP}{TP+FP}\times \frac{TP}{TP+FN}}{\frac{TP}{TP+FP}+\frac{TP}{TP+FN}} \end{array}$$

**Recall** is the amount of positive factors that were captured by the model, and is calculated after its labeling (true positive) as in Equation (6).

(6) $$\begin{array}{c} Recall\left(\%\right)=\frac{Tp}{Tp+Fn} \end{array}$$

The Equation (7) is used to assess sensitivity.

(7) $$Sensitivity\left(\%\right)=\frac{TP}{TP+FN}$$

We determined the overall effectiveness of our proposed model using the receiver operating characteristic curve (true-positive and false-positive rate) and AUC with confidence interval (CI).

## 4. Results and Analysis

The proposed method for mammogram-based breast MC prediction was developed using scientific methods. The mammogram images were obtained from the publicly available MIAS database to train and validate the proposed models. Each image was preprocessed to remove noise and enhance image quality. Each experimental test was carried out using Intel(R) Core I7, CPU 2.80 GHz and 2.80 GHz, 32 GB memory, NVIDIA GTX 1050Ti graphics card. The computation time for training and testing the proposed model was 33 min. Furthermore, this study employed Keras’ ImageDataGenerator [56] library and the OpenCV method to create batches of mammogram images with real-time data augmentation and preprocessing.

We used an Adam optimizer to train the proposed models, using an initial learning rate of 0.001 and a momentum constant of 0.9. Throughout the optimization phase, the momentum parameter, learning rate, and hidden layer neurons were modified from 0 to 1. The algorithm was trained throughout 90 epochs at a rate of 100 steps per epoch. The hyperparameters used for training are described in Table 3.

### 4.1. Microcalcification-Based ROI Detection and Segmentation

Automatic detection and extraction of MC ROI from a mammogram is a challenging task for the CAD system [53]. Previously, most clinicians extracted ROI manually, and artificial factors, such as fatigue and extensive mammogram interpretation, reduced ROI diagnostic accuracy [57]. As a result, machine learning approaches have attained significant interest in mammogram interpretation and automatic extraction of calcification areas [58]. Due to the mammogram’s low contrast and intricate breast anatomy, clinicians can observe only a small amount of information. Hence, we used image improvement techniques, such as adaptive unsharp masking [59], CLAHE, and the wavelet enhancement to emphasize the MC point while deleting irrelevant background information. This study employed three distinct strategies used for MC detection: wavelet transform, top-hat, and the combined method. The combined method based on the top-hat morphology operator and wavelet reconstruction is proposed to detect seed points to minimize false-positive rates and segmenting calcification. This study primarily employed the ROC curve, detection rate, and the average number of false detections to evaluate the microcalcification detection approach.

First, the wavelet transform eliminates the image’s low-frequency background information, only retaining the high-frequency calcification spots. After detecting microcalcification regions, different radiomic features, such as texture features, morphological, intensity histogram, and GLCM features from ROI are extracted using the wavelet transform. The wavelet reconstruction image is the thresholded using the variance method; seed points containing MC and noise are filtered out. Afterward, the top-hat operator conducts a coarse segmentation of the MC patches. The bulk of the image’s background information is removed during the ROI processing while maintaining calcification spots. After several experiments, a flat disc-shaped structural element with a radius of 5 is chosen to process the image. Figure 4a–e shows how the image’s background is effectively suppressed while the microcalcification locations are highlighted.

**Figure 5:**
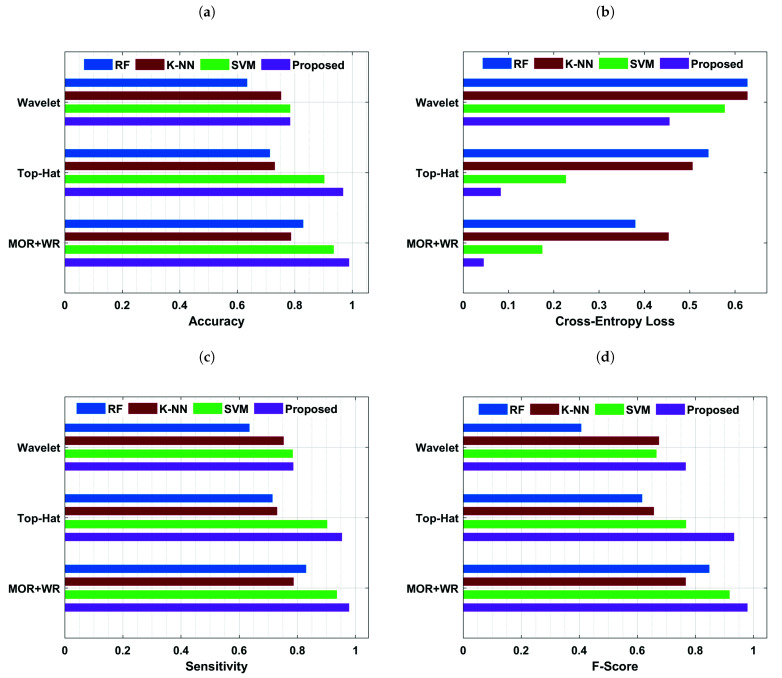
The suggested method’s performance is compared with state-of-the-art techniques in terms of (**a**) accuracy, (**b**) cross-entropy loss, (**c**) sensitivity, and (**d**) F-Score. The curves reveal that MC ROI classification through DC-ELM is excellent, which attained accuracy of 0.98, sensitivity of 0.98, and F-measure of 0.97.

Detection rate and false detection rate are important indicators for evaluating ROI extraction for breast MC points. The findings reveal that the proposed approach is beneficial for MC patches detection with a detection rate of 98.7% and a false-positive detection rate of 1.2. Contrary to this, the standard methods, such as adaptive unsharp masking, wavelet transform, and top-hat method, yielded detection rates 78.6%, 82.9%, and 93.5% and false detection 11.7%, 4.3%, and 2.9%, respectively, for predicting MC patches. Thus, the fusion of morphological top-hat operator and wavelet reconstruction efficiently suppress the image background and decrease the false positive detection rate for detecting MC patches in mammograms. By decreasing noise and strengthening weak image edges, the suggested strategy beats previous techniques in terms of detection accuracy. Despite the fact that the detection rate of the proposed approach has consistently increased, the false positive detection rate has been lowered to 1.2. Moreover, the overall performance of four proposed algorithms, based on wavelet analysis, top-hat analysis, and combined detecting approach, is plotted in Figure 5a–d.

### 4.2. Microcalcification-Based Feature Extraction and Classification

**Figure 6:**
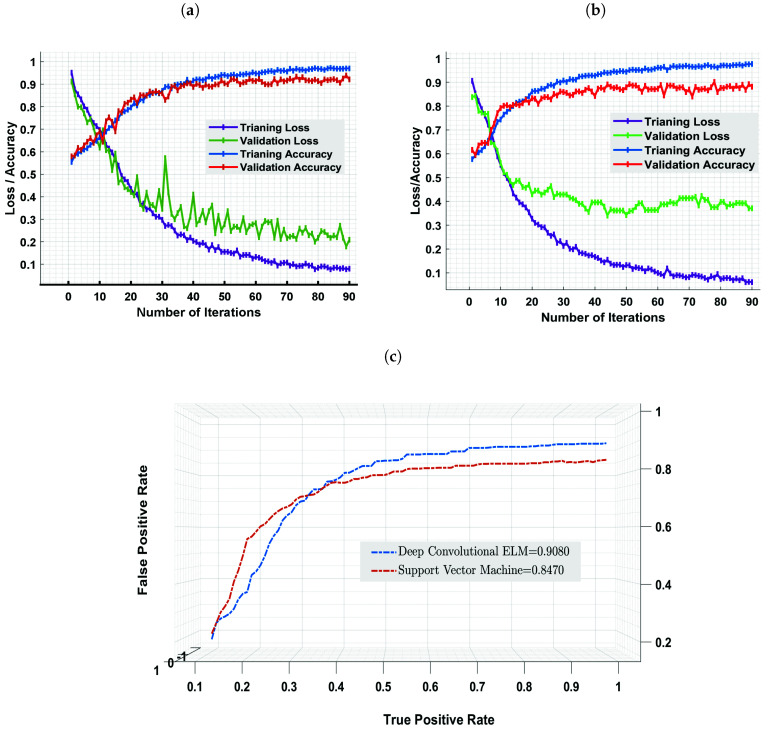
The figures represent the training and testing accuracy, cross-entropy loss, and AUC of proposed algorithm for MC classification against the 90 epochs. (**a**) DC-ELM, (**b**) SVM, (**c**) comparing the discriminative competencies of DC-ELM and SVM algorithms based on MC regions using AUC.

The current study proposed a state-of-the-art computer-aided diagnostic system using DC-ELM, K-NN, RF, and SVM to detect and classify MC ROIs from digital mammograms. The empirical results indicate that the proposed approach performs better than other models. The model’s accuracy and loss performance are shown in Figure 6a,b, which depict that the model’s accuracy increased continuously over iterations than the training loss, indicating our model trained perfectly. After the 31st epoch, the validation accuracy was constant, and the validation loss declined, revealing that our proposed approach performed well and the model was well-fitted.

Classification accuracy is measured as the ratio of correctly classified images to the total number collected for classification. As a result, the proposed approach has the benefit of enabling effective and precise accuracy for MC diagnosis. The training and validation curves (accuracy and cross-entropy loss) were computed after 90 iterations. Our proposed approach outperformed the SVM model and other models, achieving a training accuracy of 0.98 and a validation accuracy of 0.94, as plotted in Figure 6a to classify MC ROI. In comparison, the SVM model yielded a training accuracy of 0.93 and a validation accuracy of 0.88, as depicted in Figure 6b. Furthermore, other well-defined K-NN and RF algorithms achieved training and validation accuracies of 0.79, 0.83, and 0.76, 0.81, respectively. Thus, based on statistical evaluations of the accuracy indicator, the proposed model surpassed SVM, K-NN, and RF by 5%, 19%, and 15%, respectively. The overall accuracy performance of all models based on MC detection and segmentation methods is shown in Figure 5a. The generalization difference (accuracy and loss) within training and validation should be modest to avoid overfitting the model. The weight values are changed to reduce the error function and increase classification accuracy in our suggested approach.

Figure 6a illustrates the proposed model’s training and validation loss. The proposed model’s training loss decreases with each epoch, eventually reaching a minimum of 0.06 for training and 0.20 for validation at the final epoch. In contrast, SVM achieved an error rate of 0.08 for training and 0.41 for validation loss. The overall error rate of all models based on proposed MC detection methods is shown in Figure 5b. On the other hand, the proposed strategy outperforms SVM in classifying the detected MC ROI using the fusion method, with a sensitivity of 0.98 with FPi 1.2. In comparison, SVM, K-NN, and RF achieved 0.93, 0.79, and 0.83 sensitivity, respectively.

The AUC value was used to measure the accuracy of clinical diagnostic methods by comparing true-(positive and negative) and false-(positive and negative) predictions. The variations of true-positive and false-positive results of the proposed diagnostic methods and SVM classifier are illustrated in Figure 6c. The proposed model yielded the best AUC value of 0.90, comparing the 0.85, 0.83, and 0.79 achieved by the SVM, K-NN, and RF algorithms. Our model achieved a promising AUC value and significantly reduced the number of false-(positives and negatives) while creating massive true positives. The AUC curves show that the proposed model works better with fusion detection schemes because MC has higher spatial frequencies and prefers to extract high-frequency information using wavelet composition.

Table 4 compares the F-score, sensitivity, specificity, accuracy, and AUC of each approach. Figure 5c,d, illustrates that the proposed model attained F-scores of 0.98, sensitivity of 0.98, and specificity of 0.93. It was observed that our proposed model’s sensitivity, F-score, and specificity values are significantly larger than those of other models by 5%, 7%, and 2%, respectively. The projected DC-ELM approach is more competent than other state-of-the-art techniques, enabling pathologists to apply and select the quickest way with the best decisions.

All of the above in-depth assessments show that the performance of our suggested strategy was superior.

### 4.3. Comparative Analysis with Conventional Studies

To evaluate our CAD system, we compared it to earlier research using the publicly available MIAS dataset. Table 5 depicts that our technique performs much better than previous studies in detecting and classifying MC, with 0.98 accuracy and 0.98 sensitivity at a 1.2 FPi. Yang et al. [60] used the context-sensitivity technique for MC detection train on 188,521 mammograms, achieving a sensitivity of 0.87 at a 1.10 FPi. Wang et al. [22] used a deep MC detection approach and obtained a 0.87 accuracy and 0.93 sensitivity using 1000 mammograms from the private dataset. Caballero et al. [19] use an independent component analysis (ICA) to detect the MC cluster of breast cancers and obtain a sensitivity of 0.81 at 2.55 FPi by training on 200 mammogram images of DDSM datasets. Sun et al. [25] trained a deep CNN to identify breast cancer and achieved 0.50 sensitivity using 1980 breast images. Shrivastava et al. [48] proposed a seeded region growing (SRG) technique that used seed point extraction and threshold computation to extract the ROI from a mammogram image automatically. After locating the breast masses effectively, it was classified as benign or malignant and achieved 0.91 classification accuracy. Chen et al. [10] employed pre-trained DCNN ResNet architecture by fine-tuning to automatic classification using 2620 DDSM images and achieved 0.93 sensitivity.

Our model achieves a 0.98 accuracy and 0.98 sensitivity at a 1.2 FPi on 968 MIAS dataset images, which was more dominant than previous works. We found that our model is more effective than the summarized study with a high sensitivity of 0.91 [48] on MIAS, 0.93 [22] on private and 0.93 [10] on DDSM datasets, confirming that our modal performed remarkably better.

## 5. Discussions

The purpose of this study was to determine whether the presence of malignant calcifications had a predictive ability and could aid in surgical decision-making in potential therapeutic settings. It is crucial to have an accurate and well-defined method for predicting tumor response and evaluating the degree of tumor recurrence to inform the surgeon and patient about the optimal surgical approach and timing of surgery to minimize disease recurrence. Referring to the fourth BI-RADS classification, the most frequently encountered and identifying features on mammograms are MC, indicating malignant breast cancer. Calcification points were evaluated in terms of their morphology, distribution, range, density, and diameter. They were dispersed randomly and were not all associated with adjacent tissues. Radiologists analyze mammograms to identify benign breast calcification that do not need a biopsy, avoid unnecessary curing, overcome patient anxiety, and increase the patient’s survival rate. Thus, we conclude from the findings that a standard mammogram analysis before the biopsy is sufficient, and this interpretation is diagnostically helpful and critical for surgical decision-making.

This study proposes a radiomics analysis approach for detecting and classifying MC ROI in digital mammograms as benign or malignant. The proposed framework consists of four phases: preprocessing and data enhancement, MC ROI segmentation, feature extraction, and ROI classification. The model’s performance was evaluated using the MIAS dataset, which was randomly divided into training and validation sets in ratios of 80% and 20%. The input image was preprocessed applying different image filters and enhancement paradigms for ROI segmentation as depicted in Figure 4a–f. The wavelet reconstruction method increases the intensity of the calcification and blood vessels, making it easier for clinicians to observe and diagnose. Finally, the number of false-positive instances is decreased by implementing morphological calcification.

To achieve the best MC classification outcomes, we employed the optimal hyperparameter settings in our methods, which included a 32 batch size, 0.001 learning rate, a dropout of 0.5, 0.9 momenta constant, and 90 epochs, as presented in Table 3. The experimental findings reveal that the proposed model significantly outperformed with 0.98 sensitivity at a 1.2 FPi compared to other RF, K-NN, and SVM studies. As observed in Table 4, our model outperformed the SVM, K-NN, and RF models by 5% in sensitivity and AUC. The findings reveal that our radiomics analysis approach exceeds existing investigation [10,31] in MC prediction sensitivity and accuracy. Wang et al. [60] designed a context-sensitive method based on deep learning for MC detection with the sensitivity of 0.87 at a 1.10 false-positive rate. Cai et al. [18] designed a deep CNN model for automated MC classification that achieved an accuracy of 0.89 and a sensitivity of 0.86 using automatic and manual feature extraction. Another deep microcalcification detection strategy [22] obtained 0.87 accuracy and 0.93 sensitivity using 1000 mammogram images of the private dataset.

The proposed investigation can diagnose MC with accurate results and enable radiologists to detect breast cancer timely to save patients’ lives. It automatically extracts the multiscale features, such as texture, morphological, and intensity histogram features, boosting the cancer diagnosis rate and evaluation performance. The proposed model achieves a remarkable improvement in the MC cluster’s localization and true-positives using three feature vectors generated by wavelet analysis, top-hat, and combination compared to SVM, RF, and K-NN models. Our model performed significantly better than the summarized study with a highest sensitivity of 0.91 [48] on MIAS, 0.93 [22] on private and 0.93 [10] on DDSM datasets. Despite this, in the future, we will study identifying specific MCs using DCNN pre-trained architectures and classifying individual MCs on a large dataset. Moreover, we will examine our approach to increase classification accuracy on 2D and 3D mammograms.

## 6. Conclusions and Future Work

Microcalcification is a biomarker for early-stage breast cancer. Moreover, the breast’s dense anatomy and poor contrast makes it challenging to recognize MC areas, which the clinician will invariably misdiagnose. A problematic issue in the automating detection of MC clustered is the frequent appearance of false positives generated by local visual patterns that mimic MCs. We proposed a radiomics approach for automatic segmentation and classification of MC ROIs to overcome this problem. This study employed the wavelet transform and top-hat operator methods to detect and localize microcalcification localities in mammogram images. As a result, the proposed study effectively suppresses the image’s background and reduces false-positive diagnosis by combining the morphological top-hat operator with the wavelet reconstruction method. The findings indicate that the proposed classifier categorizes MC patches with high accuracy of 0.98 and sensitivity of 0.98 with a 1.2 FPi, supporting radiologists in making the proper diagnostic decisions and eliminating needless biopsies. In th future, we will focus on enhancing the mammogram’s many different characteristics and its further classification. It would be very beneficial if detection could be performed in real-time throughout the screening process.

## Figures and Tables

**Table 1:** Experimental description of the training and validation dataset.

Data Category	Benign Images	Malignant Images	Total Images
Images	536	432	968
Training set	429	346	775
Validation set	107	86	193

**Table 2:** Different data augmentation techniques used to increase the data sample.

Sr	Augmentation Techniques	Performance Values
1	Rotation	45°, 90°, 135°, 180°, 270°, 360°
2	Sharpen (lightness value)	0.5, 1, 1.5, 2
3	Crop and Pad	0.25
4	Shear (axis value)	10° (*X*-axis and *Y*-axis)
5	Flipping	Top, Bottom, Left, Right
6	Gaussian Blur (Sigma value)	0.25, 0.5, 1, 2
7	Horizontal and Vertical Shift	0.2

**Table 3:** Hyperparameter configurations.

Configuration	Values
Batch Size	32
Learning Rate	0.001
Epochs	90
Steps per epochs	100
Weight Decay	0.00005
Dropout	0.5
Momentum	0.9
Optimization function	Adam

**Table 4:** Performance evaluation comparison of proposed model with SVM, RF, and K-NN.

Classifiers	Accuracy	Loss	F-Score	Recall	Precision	Specificity	Sensitivity	AUC
RF	0.83	0.37	0.85	0.81	0.82	0.78	0.83	0.83 (CI = 75–90)
K-NN	0.79	0.38	0.77	0.73	0.79	0.76	0.79	0.79 (CI = 71–86)
SVM	0.93	0.08	0.91	0.90	0.93	0.91	0.93	0.85 (CI = 78–91)
Proposed Method	0.98	0.06	0.98	0.93	0.94	0.97	0.98	0.90 (CI = 84–95)

**Table 5:** Comparison of the proposed model finding with existing studies.

Authors	Challenges	Approaches Used	Database	ACC	SEN
[10]	Automatic MC classification	ResNet	DDSM	0.931	0.938
[18]	Characterization of MC cluster	Deep CNN	Private	0.89	0.86
[19]	Detection of MC cluster	ICA	DDSM	N/A	0.81
[22]	MC segmentation and classification	SVM, KNN	Private	0.87	0.93
[25]	Detection of breast cancer	Deep CNN	Private	0.82	0.50
[31]	MC’s ROI segmentation and classification	MIL	MIAS	N/A	0.94
[35]	Recognize and segment the breast tumor	MLP, SVM	MIAS	0.89	0.83
[39]	Mass detection and diagnosis	SVM	MIAS	0.93	N/A
[48]	Automatic segmentation of MC	SGR	DDSM	0.91	N/A
[60]	MC detection based on surround tissue	Context-Sensitive DNN	FFDM	N/A	0.87
Proposed	Detection and classification of MC	DC-ELM, SVM	MIAS	0.98	0.98

## Data Availability

MIAS datasets are publicly available from the Mammogram Image Analysis Society; source [40].

## References

[1] Mahmood T., Li J., Pei Y., Akhtar F., Imran A., Rehman K.U. (2020). A Brief Survey on Breast Cancer Diagnostic With Deep Learning Schemes Using Multi-Image Modalities. IEEE Access.

[2] Ginsburg O., Yip C.H., Brooks A., Cabanes A., Caleffi M., Dunstan Yataco J.A., Gyawali B., McCormack V., McLaughlin de Anderson M., Mehrotra R. (2020). Breast cancer early detection: A phased approach to implementation. Cancer.

[3] Maitra I.K., Nag S., Bandyopadhyay S.K. (2012). Technique for preprocessing of digital mammogram. Comput. Methods Progr. Biomed..

[4] Balleyguier C., Ayadi S., Van Nguyen K., Vanel D., Dromain C., Sigal R. (2007). BIRADS classification in mammogram. Eur. J. Radiol..

[5] Liberman L., Menell J.H. (2002). Breast imaging reporting and data system (BI-RADS). Radiol. Clin..

[6] Hadi Q., Masroor I., Hussain Z. (2019). Mammographic Criteria for Determining the Diagnostic Accuracy of Microcalcifications in the Detection of Malignant Breast Lesions. Cureus.

[7] Diyana W.M., Besar R. (2003). Automated methods in clustered microcalcifications detection module of a CAD system. J. Mech. Med. Biol..

[8] Lee S.J., Chen T., Yu L., Lai C.H. (2018). Image classification based on the boost convolutional neural network. IEEE Access.

[9] Panda R.N., Panigrahi B.K., Patro M.R. (2009). Feature extraction for classification of microcalcifications and mass lesions in mammograms. IJCSNS Int. J. Comput. Sci. Netw. Secur..

[10] Chen Y., Zhang Q., Wu Y., Liu B., Wang M., Lin Y. (2018). Fine-tuning ResNet for breast cancer classification from mammogram. The International Conference on Healthcare Science and Engineering.

[11] Craft M., Bicknell A.M., Hazan G.J., Flegg K.M. (2013). Microcalcifications detected as an abnormality on screening mammogram: Outcomes and followup over a five-year period. Int. J. Breast Cancer.

[12] Li M., Zhu L., Zhou G., He J., Jiang Y., Chen Y. (2021). Predicting the pathological status of mammographic microcalcifications via a radiomics approach. Intell. Med..

[13] Elezaby M., Li G., Bhargavan-Chatfield M., Burnside E.S., DeMartini W.B. (2018). ACR BI-RADS assessment category 4 subdivisions in diagnostic mammogram: Utilization and outcomes in the national mammogram database. Radiology.

[14] Yang L., Xu Z. (2019). Feature extraction by PCA and diagnosis of breast tumors using SVM with DE-based parameter tuning. Int. J. Mach. Learn. Cybern..

[15] Jiang Y., Metz C.E., Nishikawa R.M., Schmidt R.A. (2006). Comparison of independent double readings and computer-aided diagnosis (CAD) for the diagnosis of breast calcifications. Acad. Radiol..

[16] Watanabe A.T., Lim V., Vu H.X., Chim R., Weise E., Liu J., Bradley W.G., Comstock C.E. (2019). Improved cancer detection using artificial intelligence: A retrospective evaluation of missed cancers on mammogram. J. Digit. Imaging.

[17] Valvano G., Santini G., Martini N., Ripoli A., Iacconi C., Chiappino D., Della Latta D. (2019). Convolutional neural networks for the segmentation of microcalcification in mammogram imaging. J. Healthc. Eng..

[18] Cai H., Huang Q., Rong W., Song Y., Li J., Wang J., Chen J., Li L. (2019). Breast microcalcification diagnosis using deep convolutional neural network from digital mammograms. Comput. Math. Methods Med..

[19] Gallardo-Caballero R., García-Orellana C., García-Manso A., González-Velasco H., Macías-Macías M. (2012). Independent component analysis to detect clustered microcalcification breast cancers. Sci. World J..

[20] Lei C.q., Wei W., Liu Z.y., Xiong Q.Q., Yang C.Q., Zhu T., Zhang L.L., Yang M., Tian J., Wang K. (2019). Radiomics analysis for pathological classification prediction in BI-RADS category 4 mammographic calcifications. J. Clin. Oncol..

[21] Morin O., Vallières M., Jochems A., Woodruff H.C., Valdes G., Braunstein S.E., Wildberger J.E., Villanueva-Meyer J.E., Kearney V., Yom S.S. (2018). A deep look into the future of quantitative imaging in oncology: A statement of working principles and proposal for change. Int. J. Radiat. Oncol. Biol. Phys..

[22] Wang J., Yang X., Cai H., Tan W., Jin C., Li L. (2016). Discrimination of breast cancer with microcalcifications on mammogram by deep learning. Sci. Rep..

[23] Beham M.P., Tamilselvi R., Roomi S.M., Nagaraj A. (2019). Accurate Classification of Cancer in Mammogram Images. Innovations in Electronics and Communication Engineering.

[24] Obaidullah S.M., Ahmed S., Gonçalves T., Rato L. RMID: A novel and efficient image descriptor for mammogram mass classification. Proceedings of the Information Technology, Systems Research and Computational Physics.

[25] Sun D., Wang M., Li A. (2018). A multimodal deep neural network for human breast cancer prognosis prediction by integrating multi-dimensional data. IEEE/ACM Trans. Comput. Biol. Bioinform..

[26] de Oliveira Martins L., Junior G.B., Silva A.C., de Paiva A.C., Gattass M. (2009). Detection of masses in digital mammograms using K-means and support vector machine. ELCVIA Electron. Lett. Comput. Vis. Image Anal..

[27] Loizidou K., Skouroumouni G., Nikolaou C., Pitris C. (2020). An automated breast micro-calcification detection and classification technique using temporal subtraction of mammograms. IEEE Access.

[28] Gaikwad V.J. (2015). Detection of breast cancer in mammogram using support vector machine. Int. J. Sci. Eng. Res..

[29] Chakraborty J., Midya A., Mukhopadhyay S., Rangayyan R.M., Sadhu A., Singla V., Khandelwal N. (2019). Computer-aided detection of mammographic masses using hybrid region growing controlled by multilevel thresholding. J. Med. Biol. Eng..

[30] Liu N., Qi E.S., Xu M., Gao B., Liu G.Q. (2019). A novel intelligent classification model for breast cancer diagnosis. Inf. Process. Manag..

[31] Elmoufidi A., El Fahssi K., Jai-Andaloussi S., Sekkaki A., Gwenole Q., Lamard M. (2018). Anomaly classification in digital mammogram based on multiple-instance learning. IET Image Process..

[32] Wang Z., Li M., Wang H., Jiang H., Yao Y., Zhang H., Xin J. (2019). Breast cancer detection using extreme learning machine based on feature fusion with CNN deep features. IEEE Access.

[33] Soulami K.B., Saidi M.N., Tamtaoui A. (2016). A cad system for the detection of abnormalities in the mammograms using the metaheuristic algorithm particle swarm optimization (pso). International Symposium on Ubiquitous Networking.

[34] Guan Y., Wang X., Li H., Zhang Z., Chen X., Siddiqui O., Nehring S., Huang X. (2020). Detecting Asymmetric Patterns and Localizing Cancers on Mammograms. Patterns.

[35] Rouhi R., Jafari M. (2016). Classification of benign and malignant breast tumors based on hybrid level set segmentation. Expert Syst. Appl..

[36] Rizzi M., D’Aloia M., Castagnolo B. (2009). Computer aided detection of microcalcifications in digital mammograms adopting a wavelet decomposition. Integr. Comput.-Aided Eng..

[37] Yu S.N., Huang Y.K. (2010). Detection of microcalcifications in digital mammograms using combined model-based and statistical textural features. Expert Syst. Appl..

[38] Papadopoulosa A., Fotiadisb D.I., Likasb A. (2002). An automatic microcalcification detection system based on a hybrid neural network classifier. Artif. Intell. Med..

[39] Elter M., Held C. (2008). Semiautomatic segmentation for the computer aided diagnosis of clustered microcalcifications. Proc. SPIE Int. Soc. Opt. Eng..

[40] Suckling J.P. (1994). The mammographic image analysis society digital mammogram database. Dig. Mammo.

[41] Mahmood T., Li J., Pei Y., Akhtar F., Jia Y., Khand Z.H. Breast Mass Detection and Classification Using Deep Convolutional Neural Networks for Radiologist Diagnosis Assistance. Proceedings of the 2021 IEEE 45th Annual Computers, Software, and Applications Conference (COMPSAC).

[42] Wang Z., Xin J., Zhang Q., Gao S., Ma C., Ren J., Zhang H., Qian W., Zhu W., Zhang X. (2020). Breast microcalcifications detection based on fusing features with DTCWT. J. X-ray Sci. Technol..

[43] Al-Masni M.A., Al-Antari M.A., Park J.M., Gi G., Kim T.Y., Rivera P., Valarezo E., Choi M.T., Han S.M., Kim T.S. (2018). Simultaneous detection and classification of breast masses in digital mammograms via a deep learning YOLO-based CAD system. Comput. Methods Programs Biomed..

[44] Yu X., Pang W., Xu Q., Liang M. (2020). Mammographic image classification with deep fusion learning. Sci. Rep..

[45] Ting F.F., Tan Y.J., Sim K.S. (2019). Convolutional neural network improvement for breast cancer classification. Expert Syst. Appl..

[46] Malar E., Kandaswamy A., Chakravarthy D., Dharan A.G. (2012). A novel approach for detection and classification of mammographic microcalcifications using wavelet analysis and extreme learning machine. Comput. Biol. Med..

[47] Wang A., Liu X., Han Y., Qi C. License plate location algorithm based on edge detection and morphology. Proceedings of the 2012 7th International Forum on Strategic Technology (IFOST).

[48] Shrivastava N., Bharti J. (2020). Breast tumor detection and classification based on density. Multimed. Tools Appl..

[49] Linguraru M.G., Marias K., English R., Brady M. (2006). A biologically inspired algorithm for microcalcification cluster detection. Med. Image Anal..

[50] Jiang J., Yao B., Wason A. (2007). A genetic algorithm design for microcalcification detection and classification in digital mammograms. Comput. Med. Imaging Gr..

[51] Melekoodappattu J.G., Subbian P.S. (2020). Automated breast cancer detection using hybrid extreme learning machine classifier. J. Am. Intell. Hum. Comput..

[52] Melekoodappattu J.G., Subbian P.S. (2019). A hybridized ELM for automatic micro calcification detection in mammogram images based on multi-scale features. J. Med Syst..

[53] Mahmood T., Li J., Pei Y., Akhtar F. (2021). An Automated In-Depth Feature Learning Algorithm for Breast Abnormality Prognosis and Robust Characterization from Mammography Images Using Deep Transfer Learning. Biology.

[54] Ali A.A., Mishra S., Dappuri B. (2020). Breast cancer classification using tetrolet transform based energy features and K-nearest neighbor classifier. Recent Trends and Advances in Artificial Intelligence and Internet of Things.

[55] Fadil R., Jackson A., Abou El Majd B., El Ghazi H., Kaabouch N. Classification of Microcalcifications in Mammograms using 2D Discrete Wavelet Transform and Random Forest. Proceedings of the 2020 IEEE International Conference on Electro Information Technology (EIT).

[56] Keras Deep Learning Library for Image Data Preprocessing. https://keras.io/api/preprocessing/image/imagedatagenerator-class.html.

[57] Goudarzi M., Maghooli K. (2018). Extraction of fuzzy rules at different concept levels related to image features of mammogram for diagnosis of breast cancer. Biocybern. Biomed. Eng..

[58] Shi P., Zhong J., Rampun A., Wang H. (2018). A hierarchical pipeline for breast boundary segmentation and calcification detection in mammograms. Comput. Biol. Med..

[59] Polesel A., Ramponi G., Mathews V.J. Adaptive unsharp masking for contrast enhancement. Proceedings of the International Conference on Image Processing.

[60] Wang J., Yang Y. (2018). A context-sensitive deep learning approach for microcalcification detection in mammograms. Pattern Recognit..

